# Efficient Generation of RNA Secondary Structure Prediction Algorithm Under PAR Framework

**DOI:** 10.3389/fpls.2021.830042

**Published:** 2022-01-21

**Authors:** Haihe Shi, Xiaoqian Jing

**Affiliations:** School of Computer and Information Engineering, Jiangxi Normal University, Nanchang, China

**Keywords:** algorithm component library, feature modeling, PAR platform, RNA secondary structure prediction algorithm, generative programming

## Abstract

Prediction of RNA secondary structure is an important part of bioinformatics genomics research. Mastering RNA secondary structure can help us to better analyze protein synthesis, cell differentiation, metabolism, and genetic processes and thus reveal the genetic laws of organisms. Comparative sequence analysis, support vector machine, centroid method, and other algorithms in RNA secondary structure prediction algorithms often use dynamic programming algorithm to predict RNA secondary structure because of their huge time and space consumption and complex data structure. In this article, the domain of RNA secondary structure prediction algorithm based on dynamic programming (DP-SSP) is analyzed in depth, and the domain features are modeled. According to the generative programming method, the DP-SSP algorithm components are interactively designed. With the support of PAR platform, the DP-SSP algorithm component library is formally realized. Finally, the concrete algorithm is generated through component assembly, which improves the efficiency and reliability of algorithm development.

## Introduction

RNA is one of the important macromolecules in organisms and plays an important role in protein synthesis, cell differentiation, metabolism, and genetic process. Especially in HIV and other viruses, genetic information is carried directly by RNA rather than DNA (Jiang et al., 2002). To better analyze the role of RNA molecules in the life process, it is necessary to understand the molecular structure of RNA. The molecular structure of RNA can be divided into three levels (Peter and Rdf, 2000; Yang, 2013), namely, primary structure, secondary structure, and tertiary structure. Primary structure refers to a sequence composed of four bases (A, U, C, and G) of RNA. The secondary structure is a two-dimensional planar structure formed by pairing partial bases on the basis of the primary structure, and tertiary structure is a three-dimensional structure formed by folding on the basis of secondary structure (Yu, 2009; Huang et al., 2014). It has been proved that RNA tertiary structure plays a decisive role in RNA function, but the prediction of RNA tertiary structure largely depends on the prediction of secondary structure (Shuaimin, 2019; Bowen, 2021; Zhaokui and Yuanchao, 2021). Therefore, RNA secondary structure prediction is an important and hot issue in the field of RNA research. Since 1980s, RNA secondary structure prediction algorithms have emerged one after another, which can be roughly divided into two categories: one is comparative sequence analysis method, such as Stochastic Context-free Grammar (SCFG) model (Dowell and Eddy, 2004) and Covariance Model (CM) (Eddy and Durbin, 1994); and the other is the prediction method based on dynamic programming. Typical examples include the maximum base pairs algorithm proposed by Nussinov of Weizmann Institute of Science (Nussinov et al., 1978); the minimum free energy algorithm designed by Zuker of Division of Biological Sciences, National Research Council of Canada (Zuker and Stiegler, 1981); the partition function algorithm established by McCaskill of Max-Planck lnstitut fur Biophysikalische Chemie (McCaskill, 1990); and the helix-based prediction algorithm studied by the team of Harbin Institute of Technology (Xia, 2008). Because the former requires a large number of homologous RNA sequences in advance to predict, the time and space complexity of the algorithm is particularly high, and long sequences cannot be analyzed well, people often use dynamic programming model to predict RNA secondary structure.

Most of the existing RNA secondary structure prediction algorithms based on dynamic programming focus on the optimization of specific steps of specific algorithms, such as accelerating the execution speed of algorithms through parallelization technology, and the optimization results will have different effects on different sequences. In addition, the complexity of the RNA secondary structure prediction problem and the diversity of algorithm design strategies make the reliability of the algorithm development difficult to guarantee and the development cost high, which is not convenient for researchers to study.

In this article, the domain of RNA secondary structure prediction algorithm based on dynamic programming (DP-SSP) is regarded as a specific domain. Through in-depth analysis of the DP-SSP domain, the commonness and differences of the domain are extracted, and the generic algorithm component library in the DP-SSP domain is designed by combining domain engineering, feature modeling, formal method PAR, and other related technologies. Then, the abstract generic programming language Apla is used to formalize the implementation. Finally, using the program conversion system of PAR platform, the components of the component library are manually assembled according to the configuration knowledge and generate a specific algorithm, thereby improving the development efficiency of the RNA secondary structure prediction algorithm and ensuring the reliability of the algorithm development.

The section “Materials and Methods” introduces related theories and methods of domain engineering, generative programming (GP), formal method PAR, and so on. The section “Domain Analysis and Abstraction of RNA Secondary Structure Prediction Algorithm” analyzes the domain of RNA secondary structure prediction algorithm domain, establishes the domain feature model of DP-SSP, and implements it by using the generic abstract programming language Apla in the formal method PAR, finally establishing a high abstract component library based on abstract data type (ADT). The section “Results” shows the process of developing Zuker algorithm based on the component library and gives the experimental results of the algorithm and the comparison with other algorithms. Finally, in the section “Discussion,” the full text is summarized and prospected.

## Materials and Methods

### PAR Framework

PAR framework (Jinyun, 1993, 1998; Xue, 1997, 2015; Shi and Xue, 2009; Wang and Xue, 2009) includes a practical formal method and corresponding support platform. PAR platform includes requirements design language SNL, algorithmic modeling language Radl, abstract programming language Apla, and a series of conversion rules and automatic conversion tools. PAR focuses on the design and implementation of algorithms, supports most of the current mainstream algorithm design technologies, includes a new development strategy of loop invariant, and implements the distributed transaction processing system and relational database mechanism. By using PAR method to develop algorithms, we can have a deeper understanding of the algorithm and avoid the difficulty of selecting the design method. The agile generic mechanism is one of the important features of PAR. Regardless of the data type, data value, calculation operation, or user-defined ADT, it can be a generic parameter. Apla can directly use ADTs and abstract processes programming. It not only has the advantages of concise mathematical language but also has the characteristics of expressing unambiguity. Due to its high abstraction, Apla is very suitable for describing abstract algorithm programs. The following describes the implementation mechanism and constraint mechanism of Apla generics:

(1) Apla includes the concepts of type variables, type domains, operation variables, operation domains, ADT variables, and ADT domains. It uses sometype, someaction, and someadt to represent type domain, operation domain, and ADT domain, respectively, and implements the parametric operation of type, function, program, and custom ADT. In the instantiation process, actual parameters that meet the relevant attribute conditions can be passed in to implement different program units.
(2) Generic constraints describe the types and composition of generic parameters in detail. The implementation of generic constraint mechanism can greatly improve the reliability of generic programming, which is one of the necessary conditions for the real implementation of generic programming. PAR platform implements relevant generic constraints on generic parameters such as basic data type, ADT type and subroutine type, and proposes corresponding constraint description, matching, and detection mechanisms, which are still being improved.

**FIGURE 1:**
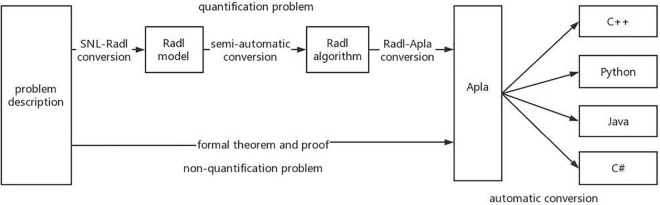
The development algorithm flow of PAR method.

In addition, PAR platform also supports the transformation of Apla into executable high-level programming languages such as C++, Java, C#, and Delphi, which has a good support for the development of components. PAR has established two ways to formalize the way to develop programs, and its platform architecture is shown in Figure 1. The first is that for quantitative problems, the PAR method can convert the SNL requirement model into the Radl specification model, then into the Radl algorithm model, and further into the Apla abstract program model, and finally into a high-level language program that can be run directly. The second way is that for nonquantitative problems, we can manually design the Apla program directly through the SNL requirement model, supplemented by the corresponding formal proof, and then convert the Apla program into an executable program.

### Domain Engineering

Domain engineering (Neighbors, 1989; Li et al., 1999; Hu and Wei, 2008) is the basic process of software reuse, and its purpose is to acquire and use reusable resources in a specific domain to develop high-quality software efficiently and at low cost. Domain engineering mainly analyzes, designs, and implements the domain. Domain analysis includes a series of activities such as system scope definition, domain requirement definition, and related terminology analysis, and finally, the results are reflected in the domain model. The domain design completed the architecture design of the system family in the domain, identified the corresponding functions and related constraints, and made plans for the subsequent implementation process. Domain implementation uses appropriate technology to complete the development of reusable resources such as architecture and components. These three stages adopt the idea of gradual refinement in practical application and modify and improve the completed results at any time according to changes in requirements.

Domain analysis is the basis of domain engineering. The generated domain model affects the quality of subsequent work. It usually adopts a combination of top-down and bottom-up analysis to repeat domain analysis activities. Top-down analysis takes into account the needs of future systems in the domain, while the bottom-up analysis mainly considers the existing systems and the reusable resources accumulated by previous development. After years of efforts by researchers, many methods have been used in domain analysis, such as organization domain modeling (ODM), object-oriented analysis (OOA), and feature-oriented domain model (FODM) (Wartrk, 1999; Chastek et al., 2001). To carry out domain analysis activities efficiently, Zhang and Mei (2003) put forward a feature modeling method FODM, which focuses on the characteristics of services, functions, and behaviors in the domain and discusses the presentation form of a feature model and its detailed modeling process.

### Generative Programming

Generative programming (Czarnecki et al., 2000; Fan and Zhang, 2005) is a new type of software paradigm, which accords with the idea of software reuse. It uses components and makes software products in an automated way, which is of great significance to solve the “software crisis.” There are two steps to implement GP. The first is to change the current software development mode into the development of software system families and develop generators to automatically assemble components. GP is an example of domain engineering application, which needs to make full use of existing domain knowledge to complete component development in corresponding domain. Finally, generator is used to develop new software in the field by means of component assembly, without the need to follow the steps of software engineering to start programming from scratch.

The purpose of GP is to realize the production automation of components and applications, and the key part of GP is to establish domain models for system families. The generative domain model consists of three parts: problem space, solution space, and corresponding configuration knowledge. The problem space mainly includes the needs of application programmers and users for the system, and the requirements are generally described by the concepts and characteristics of the program; the solution space includes the relevant components that can solve the demand problem and the combination mode of each component, and it is required to achieve the maximum composability, and the redundancy between the combinations must be minimized, and the highest reusability of the components must be achieved as much as possible. The configuration knowledge is the mapping relationship between the problem space and the solution space, which avoids the occurrence of illegal feature combination and sets the default parameters and rules of features. Configuration knowledge is the mapping relationship between the problem space and the solution space, which avoids the illegal feature combination and sets the default parameters and rules of features.

### Concepts Related to RNA Secondary Structure Prediction

RNA sequence: RNA sequence refers to the primary structure S = S_1_S_2_S_3_…S_n_ of RNA, where *S*_*i*_ ∈ (*G, C, U, A*), 1 ≤ *i* ≤ *n*.

Base pair: If (S_i_, S_j_) ∈ {*GC*, *AU*, *UG*}, 1 ≤ *i* < *j* ≤ *n*, then (S_i_, S_j_) constitutes a base pair.

RNA secondary structure: A set of base pairs.

RNA secondary structure prediction: Input an RNA sequence, predict the secondary structure through some algorithm, and follow the following rules in the prediction process:

(1) A base cannot be paired with two or more bases at the same time. That is, there are base pairs (S_*i*_, S_*j*_) and (S_*k*_, S_*l*_). If i = k, then j = l.
(2) If i < g < j < h or g < i < h < j, (S_*i*_, S_*j*_) and (S_*g*_, S_*h*_) cannot appear in the secondary structure, that is, pseudoknot cannot appear in the secondary structure.
(3) If there are base pairs (S_*i*_, S_*j*_), then |*j*−*i*| ≥ 4, that is, the length of hairpin loop structure should be ≥ 4.

Hairpin loop: A structure consisting of one base pair (S_*i*_, S_*j*_) and all unpaired bases closed by it.

Stem: A structure composed of two adjacent base pairs (S_*i*_, S_*j*_), (S_*i* + 1_, S_*j*–1_).

Bulge loop: It is composed of two base pairs (S_*i*_, S_*j*_) and (S_*k*_, Sl), and the two base pairs are adjacent at one end and not adjacent at the other end (k = i + 1, k < l < j − 1or l = j − 1, i + 1 < k < l).

Interior loop: It is composed of two base pairs (S_*i*_, S_*j*_) and (S_*k*_, S_*l*_), and the two base pairs are not adjacent at both ends (i + 1 < k < l < j = 1).

Multibranched loop: A structure closed by three or more base pairs.

### Domain Analysis and Abstraction of RNA Secondary Structure Prediction Algorithm

Here, we briefly analyze the core ideas of three typical dynamic programming algorithms.

(1) Nussinov algorithm
  Given a sequence s, when the i-th base S_*i*_ is paired with the j-th base S_*j*_ in S, θ(*i, j*) = 1, otherwise θ(*i, j*) = 0. M(i, j) is used to represent the maximum matching base logarithm on the subsequence S_*ij*_, and its value can be iterated by the following formula:

(1) $$M\left(i,j\right)=\text{Max}\left\{ \begin{array}{c} M\left(i+1,j\right)\\ M\left(i,j-1\right)\\ M\left(i+1,j-1\right)+\delta \left(i,j\right)\\ \mathrm{Max}\left(M\left(i.k\right)+M\left(k+1,j\right)\right) \end{array}\right.$$

in which i ≤ k ≤ j, when i = 1, 2, 3, …, n, M(i, i) = 0. When i = 2, 3, 4, …, n, M(i − 1, i) = 0.
The four terms in formula (1) correspond to the possible pairing between the i-th base and the j-th base in the sequence:
  ➀ S_*i*_ does not participate in base pairing, then the maximum number of base pairing in interval (i, j) is equal to the maximum number of base pairing in interval (i + 1, j).
  ➁ S_*j*_ does not participate in base pairing, then the maximum number of base pairing in interval (i, j) is equal to the maximum number of base pairing in interval (i, j = 1).
  ➂ S_*i*_ is paired with S_*j*_, and the maximum number of base pairs in interval (i, j) is equal to the maximum number of base pairs in interval (i + 1, j = 1) plus 1.
  ➃ S_*i*_ is paired with base S_*k*_ in interval (i, j), then the maximum number of base pairings in interval (i, j) is equal to the number of pairings in interval (i, k) plus the pairing number of interval (k + 1, j). Take k = i, k = i + 1, k = i + 2,…, j in turn, then the maximum number of base pairings in interval (i, j) is equal to the one with the largest number.

Each iteration takes the maximum of the above four cases, and the value of M(1, n) is the maximum number of base pairs. The secondary structure of sequence s can be obtained by backtracking from W(1, n).

(2) Zuker algorithm
  Give a sequence s, fragment S_*ij*_ represents the subsequence from the i-th base to the j-th base in the s sequence, where 1 ≤ i ≤ j ≤ n. W(i, j) is the minimum free energy of all RNA secondary structures composed of subsequence S_*ij*_ (whether S_*i*_ and S_*j*_ are paired or not), V(i, j) is the minimum free energy of RNA secondary structure formed by pairing S_*i*_ and S_*j*_. The calculation process of W(i, j) and V(i, j) is shown in formulas (2)–(6) as follows:

(2) W(i,i)=0

(3) W(i,j)=V(i,j)=ifj-i<4

(4) V(i,j)=ifiandjarenotpaired.

(5) V(i,j)={E1=EH(i,j)E2=Es(i,j;i+1,j-1)+V(i+1.j-1)E3=min{EL(i,j;i′,j′)+V(i,′j)′},i<i<′j′<j,(i-′i)+(j-j)′>2E4=min⁡{Wm(i+1,k)+Wm(k+1,j-1)},i<k<j-1

(6) W(i,j)=min{V(i,j)W(i+1,j),W(i,j-1),min{W(i,k)+W(k+1,j)},i<k<j-1},j-i≥4

E_H_(i,j) in formula (5) represents the minimum free energy corresponding to the hairpin loop structure formed by pairing base S_*i*_ and base S_*j*_, E_*s*_ represents the minimum free energy corresponding to the stem structure formed by the pairing of base S_*i*_ and base S_*j*_, E_L_ represents the minimum free energy corresponding to the interior-loop or bulge-loop structure formed by the pairing of base S_*i*_ and base S_*j*_, and E_4_ represents the minimum free energy corresponding to the multibranched loop structure formed by the pairing of base S_*i*_ and base S_*j*_.
By using formula (6) to iterate continuously, w(1, n) is the minimum free energy of sequence s. The secondary structure of sequence s can be obtained by backtracking from W(1, n).

(3) Helix-based algorithm
  Given an RNA sequence s, all possible stem regions were calculated by using the INN-HB energy model. E_*i*_,_*j*_ represents the minimum free energy of the subsequence S_*ij*_, and its value can be obtained by using formula (7).

(7) $$E_{i,j}=\left\{ \begin{array}{c} E_{init}\\ E\left(H_{i,j,k}+E_{i+k,j-k}\right)\\ min\left[E_{i,k}+E_{k+1,j}\right] \end{array}\right.$$

Equation (7) corresponds to three situations: ➀ If j − i < 8, then E_*i,j*_ = E_*init*_ = 0; ➁ Otherwise, search the stem regions, if there is a stem region H_*i,j,k*_ starting with the i-th base and ending with the j-th base, and H_*i,j,k*_ + E_*i* + *k,j*–*k*_ < E_*i,j*_, then E_*i,j*_ = E(H_*i,j,k*_ + E_*i* + *k,j*–*k*_). ➂ For each k(i < k < j), if E_*i,k*_ + E_*k* + 1,j_ < E_*i,j*_, then E_*i,j*_ = E_*i,k*_ + E_*k* + 1,j_. When E_1,*n*_ is calculated, the minimum free energy of RNA is found, and the secondary structure with the minimum free energy can be found by backtracking.

**FIGURE 2:**
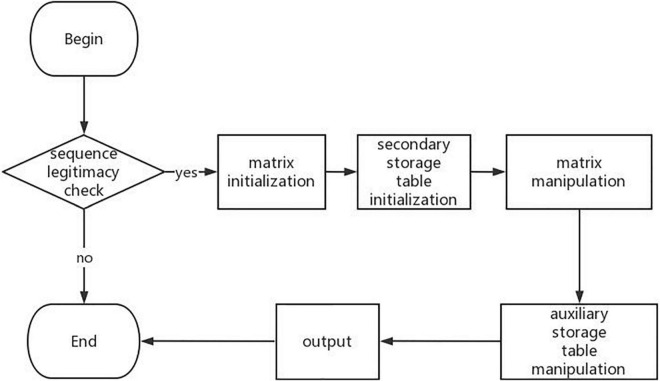
Flowchart of RNA secondary structure prediction algorithm.

By further analyzing a large number of RNA secondary structure prediction algorithms based on dynamic programming, we can know that the process of RNA DP-SSP can be summarized as shown in Figure 2.

Next, we analyze the DP-SSP domain with FODM method, and consider the Service, Function, and Behavior in the DP-SSP domain to build a feature model. The scope of the algorithm field is limited to an algorithm form with dynamic programming as the main strategy and RNA secondary structure prediction as the main prediction method in the field of RNA function analysis. RNA secondary structure prediction is the core service in this field. The user-defined RNA secondary structure prediction algorithm is realized by controlling the prediction mode, the execution priority, and the combination mode between algorithm features in the process of RNA secondary structure prediction.

(1) Sequence validity check (seq_check) is a preprocessing operation that must be performed on the input sequence before each algorithm runs, which is regarded as a common function.
(2) All algorithms in this field need to build matrices and auxiliary storage tables to store data and also need to operate matrices and auxiliary storage tables, so matrix manipulating (matrix_mani) and auxiliary storage table manipulating (auxiliary_storage_mani) are the required components. Further analysis shows that for auxiliary_storage_mani, the auxiliary storage mode (auxiliary_mode) is its behavior characteristic, and there are mainly the following ways: auxiliary matrix (matrix_op), auxiliary stem pool (stem pool_op), and auxiliary free energy parameter (free energy_op).
(3) In this field, the dynamic programming idea is used to predict the RNA secondary structure. Different RNA secondary structure prediction algorithms can be obtained by selecting different dynamic programming strategies. Therefore, dynamic programming pattern selection is regarded as a common component in this field. There are four behavior patterns: based on maximizes base pairs (Nussinov_op), based on minimizes free energy (Zuker_op), based on helix-based (helix_op), and based on partition function (partition function_op).
(4) Output function (result_op) as a common function in the field, it has two output modes: matching logarithmic output (pairing number_op) and matching interval output (pairing interval_op). Among them, the matching interval needs backtracking (backtrack) and remembering the source of elements (element source). Therefore, tracing back and remembering the source of elements are optional components.

**FIGURE 3:**
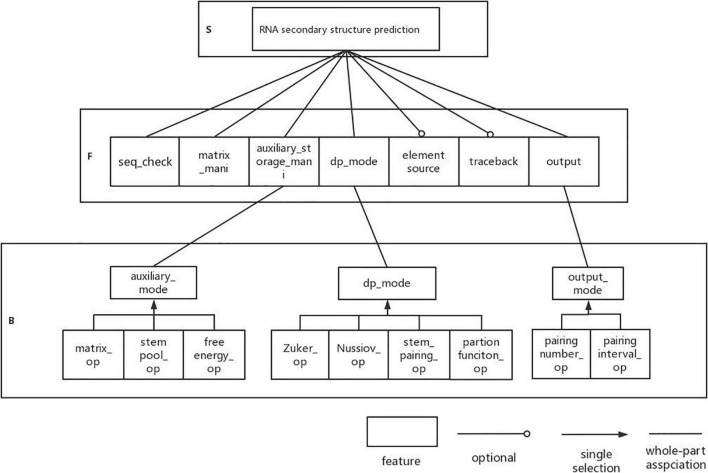
DP-SSP feature model.

The established feature model is shown in Figure 3.

A complete domain feature model also needs the interaction between features. In the feature model, the interaction between features is mainly reflected by the constraints and dependencies between features. Therefore, for the feature model established above, we design the feature interaction model in DP-SSP domain.

**FIGURE 4:**
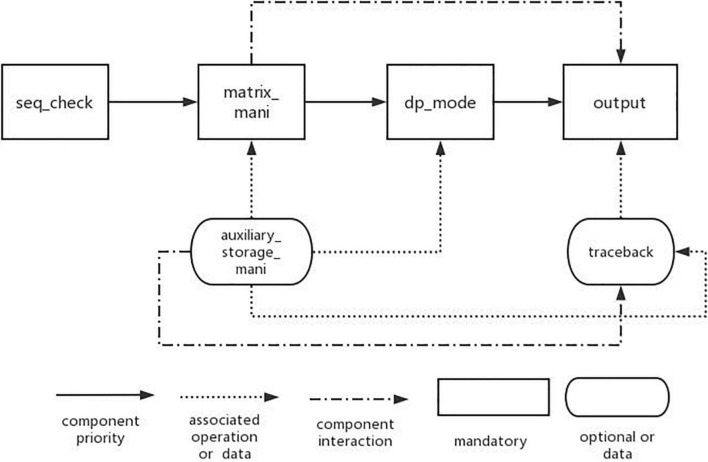
Feature interaction model of algorithm components.

Through the establishment of DP-SSP feature model, it is analyzed that the algorithm mainly includes three change process features: matrix_mani, dp_mode, and output. In addition, the input of the algorithm in this field is gene sequence, so it is necessary to check the legality of the sequence information before the algorithm is executed. Therefore, the main components in this field are seq_check component, matrix_mani component, dp_mode component, and output component. Other features in the feature model are used as auxiliary components, and the interaction model of components is established according to the dependencies between components, as shown in Figure 4.

Among them, the nodes connected by the solid line represent the basic features that must be contained in the DP-SSP field. The direction indicated by the arrow indicates the execution priority of the four features from high to low. The arrow with dotted line represents the associated operations required in the algorithm assembly process. For example, when we choose dynamic programming mode or perform matrix operations, we need to use the information of auxiliary storage table operations. The dotted line indicates the interaction between the two features in the process of algorithm execution. For example, matrix operation features need to be used in result output or backtracking.

Here, we further analyze the feature model and algorithm component interaction model in the above DP-SSP field and package them into two ADT components and an RNA secondary structure prediction algorithm component. With the advantages of high abstraction, good support for ADT, easy formal derivation, and correctness verification of Apla program, the DP-SSP model is formally designed and implemented based on Apla code.

(1) Matrix-type component
*define ADT matrix_mani (sometype elem);*
*type matrix_mani = private;*
*var:*
*matrix:list(array[0…n,elem])*
*aux:auxiliary_storage_mani*
*procedure apply_memory (m: matrix _ mani;length:integer);*
*procedure init_matrix(proc initial(); m:matrix_mani;matrix: list (array[0…n, elem]));*
*procedure setvalue (m:matrix_mani; matrix:list(array [0…n,elem]); i:integer; j:integer; aux:auxiliary_storage_mani);*
*function getvalue(m: matrix_mani; matri x:list(array [0…n,elem]);i:integer;j:integer):elem*
*function the_last_element():elem;*
*procedure traceback(m: matrix_mani; matrix:list(array [0…n,elem]); aux: auxiliary _ storage_ mani);*
*procedure output(m:matrix_mani; matrix: list(array [0…n,elem]);aux:auxiliary_storage_mani);*
*enddef;*

The generic ADT named *matrix_mani* contains a type parameter *elem*, which can accept character type or other types of data. *Type matrix_mani = private* is the storage space description, which is used to indicate that the storage space used by the custom ADT is private. *Apply_memory (m:matrix_mani;length:integer)* is used to dynamically allocate memory space for *matrix_mami* according to the value of integer variable length; *init_matrix(proc initial(); m:matrix_mani; matrix: list (array[0…n,elem]))* has a generic process parameter. Different process parameters can be passed in to instantiate different matrices. The functions of *Procedure setvalue (m:matrix_mani;matrix:list (array[0…n,elem]); i:integer; j: integer; aux: auxiliary_ storage_mani)* and *getvalue (m:matrix_mani; matrix: list (array[0…n,elem]); i:integer; j:integer):elem* are to set element values and obtain element values, respectively; *the_last_element():elem* indicates the last element in *matrix_mani*, *i(0 ≤ i ≤ length),j(0 ≤ j ≤ length)* indicates the subscript of the corresponding element, and *length* means the length of RNA sequence. *Traceback (m:matrix_mani; matrix: list (array[0…n,elem]); aux:auxiliary_storage_mani)* means to backtrack the results. *Output (m:matrix_mani; matrix: list (array[0…n,elem]);aux:auxiliary_storage_mani)* is used to output the final result. By default, it outputs the interval of base pairing.

(2) Auxiliary storage table-type component
*define ADT auxiliary_storage_mani(someproc inilization (sometype:elem);n:integer);*
*type auxiliary_storage_mani = private;*
*procedure set_value(a: auxiliary_ storage _mani;i:integer; j:integer);*
*function get_value(a: auxiliary_storage_ mani;i:integer;j: integer):elem;*
*procedure traceback(a: auxiliary_storage _mani);*
*enddef;*

The ADT contains a process generic parameter *someproc initialization_ auxiliary()* and an integer parameter *n* so that generic programs can support instantiation of different dynamic programming patterns. T*ype auxiliary_storage_mani = private* is the storage space description, which is used to indicate that the storage space used by the custom ADT is private. The functions of *Procedure set_value (a: auxiliary_ storage_ mani; i:integer;j:integer)* and *Function get_value(a: auxiliary_storage_mani; i: integer; j:integer): elem* are to set element values and obtain element values, respectively. *Procedure traceback(a:auxiliary_ storage_ mani)* means backtracking *auxiliary_storage_mani*.

(3) Secondary structure prediction algorithm component
*procedure RNA_prediction(m: matrix_mani; a:auxiliary_ storage_mani)*
*begin*
*m.apply_memory(m,length);*
*m.init_matrix();*
*do*
*i,j ≤ length*
→
*m.setvalue(m.matrix,i,j,a);*
*od;*
*m.traceback(m,a);*
*M.output(m,matrix,a);*
*end;*

The algorithm component contains two generic parameters *m* and *a*; corresponding to types *matrix_mani* and *auxiliary_storage_mani*, respectively, users can get different RNA secondary structure prediction algorithms by passing in different ADT parameters.

### Development of Zuker Algorithm Based on ADT

*program Zuker;*
*Procedure Zuker_auxiliary_initialization(char);*………… ..➀
*var*
*i:integer;*
*length:integer;*
*symbol:char;*
*begin*
*open(D:\Zuker\sourcedata.txt)*
*foreach(i = 0;i< = length;i++)*
*……*  *//Code segment, omitted.*
*end;*
*ADT Zuker_auxiliary:new auxiliary_ storage_mani(Zuker_ auxiliary_initialization,4);*……………➁
*ADT Zuker_ matrix_mani:new matrix_ mami (double);*…………………………………………➂
*Zuker_RNA_prediction():Procedure RNA_prediction (Zuker_ matrix_mani; Zuker_auxiliary);*…➃
*Var:*
*m:Zuker_ matrix_mani*
*a:Zuker _ auxiliary*
*Begin //*  *Main program code*………………………………………………➄
*open(D:\Zuker\sourcedata.txt)*
*foreach(i = 0,j = 0;i <=length, j< = length;i++,j++)*
*……*  *//Code segment, omitted.*
*Zuker_RNA_prediction(m,a);*
*end;*

Code block ➀ indicates the dynamic planning mode of Zuker algorithm, ➁ indicates the instantiation of auxiliary storage table of Zuker algorithm, ➂ indicates the main matrix of instantiating Zuker algorithm, ➃ indicates the implementation of prediction code of Zuker algorithm, and ➄ the following code blocks are the main programs. As the above Apla program cannot be run directly, we use Apla-C++ converter in PAR platform to convert Apla program into C++ program for experimental comparison.

## Results

**FIGURE 5:**
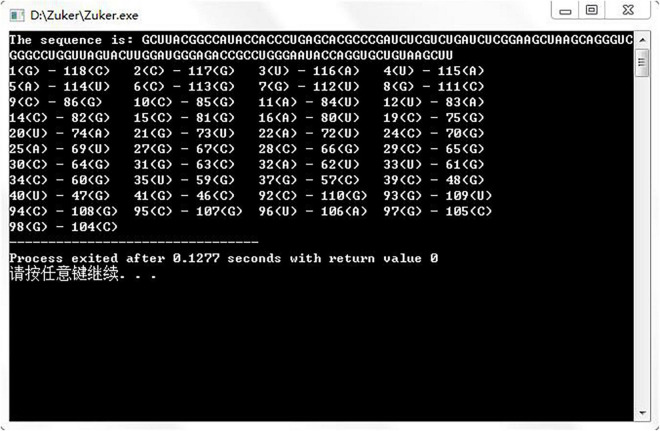
Experimental results of assembly algorithm.

Gutell laboratory provided a large number of real secondary structures of RNA, so we downloaded six real RNA sequences from http://www.rna.icmb.utexas.edu/ to run the assembly algorithm. Figure 5 shows the prediction result of an RNA sequence named d.5.e.C.carpio. Table 1 shows the comparative experiments of our assembled algorithm with partition function and Nussinov algorithm in this field.

**TABLE 1:** Experimental results of different input sequences.

RNA name	Sequence length	Partition function algorithm			Nussinov algorithm			Assembly algorithm		
		X	Y	MCC	X	Y	MCC	X	Y	MCC
d.5.e.C.carpio	120	0.66	0.68	0.67	0.64	0.59	0.61	0.61	0.63	0.62
a.I1.e.L.dispersa	218	0.49	0.44	0.46	0.46	0.43	0.44	0.62	0.63	0.62
a.I1.e.P.inouyei	380	0.53	0.59	0.56	0.36	0.29	0.32	0.68	0.65	0.66
a.I1.e.Staurastrum.sp	423	0.45	0.49	0.47	0.30	0.29	0.30	0.53	0.51	0.52
b.I1.e.H.rubra	543	0.42	0.29	0.35	0.19	0.17	0.17	0.51	0.46	0.48
a.16.m.L.tarentolae	670	0.16	0.21	0.18	0.15	0.18	0.16	0.23	0.24	0.23H

At present, researchers often use sensitivity (X), specificity (Y), and Matthews correlation coefficient (MCC) to measure the prediction accuracy of the algorithm. Sensitivity refers to the percentage that the real base pairs in the secondary structure are correctly predicted. Specificity refers to the percentage of correct prediction among all predicted base pairs. It is difficult to take both into account in general prediction methods, so researchers often use MCC to compromise. The calculation formulas are as follows:


(8) $$X=\frac{TP}{TP+FN}$$

(9) $$Y=\frac{TP}{TP+FP}$$

(10) $$MCC=\frac{TP*TN-FP*FN}{\sqrt{\left(TP+FP\right)\left(TP+FN\right)\left(FN+FP\right)\left(TN+FN\right)}}$$

where TP represents the number of base pairs correctly predicted; FN indicates the logarithm of base pairs in all real structures that are not predicted; and FP represents the predicted logarithm of base pairs that do not exist in the real structure. The value range of MCC is −1 (TP = TN = 0, completely wrong) to 1(FP = FN = 0, absolutely right). Sometimes, for convenience, people often simplify formulas (10) and (11) to evaluate the prediction results.


(11) $$MCC=\sqrt{XY}$$

In this experiment, formulas (8), (9), and (11) are used to evaluate the assembly algorithm.

According to the data in Table 1, when the sequence length is 120, 218, 380, 423, 543, and 670, respectively, the algorithm assembled in this article can obtain a better result. The X parameter, Y parameter, and MCC parameter are not inferior to the other two popular RNA secondary structure prediction algorithms. This shows that the algorithm generated by assembly has certain practicability. In addition, using the formal method PAR to develop the algorithm can also improve the development efficiency and reliability of the algorithm, which is convenient for researchers to maintain and optimize. Users only need to select different components for assembly according to the configuration knowledge to generate different specific algorithms. With the continuous expansion of DP-SSP component library, we are expected to assemble a more efficient new RNA secondary structure prediction algorithm.

## Discussion

RNA secondary structure prediction is a hot research direction in bioinformatics, and its implementation algorithm has been widely studied. Because of the flexibility of its algorithm design strategy and the complexity of the problem, this kind of algorithm is full of diversity and complexity. In this article, the GP technology is used to deeply analyze the domain of RNA DP-SSP, find out the general features and variable features, design a highly abstract program component based on Apla language by using the formal method PAR, and use PAR platform to assemble and generate Zuker algorithm, thus improving the reliability and reusability of the algorithm component and reducing the development cost.

## Data Availability Statement

The original contributions presented in the study are included in the article/supplementary material, further inquiries can be directed to the corresponding author.

## Author Contributions

HS instructed the whole research work and revised the manuscript. XJ designed the experiments and developed the original manuscript. Both authors contributed to the article and approved the submitted version.

## Conflict of Interest

The authors declare that the research was conducted in the absence of any commercial or financial relationships that could be construed as a potential conflict of interest.

## Publisher’s Note

All claims expressed in this article are solely those of the authors and do not necessarily represent those of their affiliated organizations, or those of the publisher, the editors and the reviewers. Any product that may be evaluated in this article, or claim that may be made by its manufacturer, is not guaranteed or endorsed by the publisher.
